# Glottis Recognition Software Development Using Artificial Intelligence

**DOI:** 10.7759/cureus.61464

**Published:** 2024-05-31

**Authors:** Yasushi Masumori, Soichiro Inoue, Yusuke Seino, Mamoru Konishi, Hiroyuki Nishikawa

**Affiliations:** 1 Anesthesiology, St. Marianna University School of Medicine, Kawasaki, JPN; 2 Artificial Intelligence, Focus Systems Corporation, Tokyo, JPN

**Keywords:** automatic endotracheal intubation, vocal cord recognition, video laryngoscope, endotracheal intubation, artificial intelligence

## Abstract

The use of video laryngoscopes has enhanced the visualization of the vocal cords, thereby improving the accessibility of tracheal intubation. Employing artificial intelligence (AI) to recognize images obtained through video laryngoscopy, particularly when marking the epiglottis and vocal cords, may elucidate anatomical structures and enhance anatomical comprehension of anatomy. This study investigates the ability of an AI model to accurately identify the glottis in video laryngoscope images captured from a manikin. Tracheal intubation was conducted on a manikin using a bronchoscope with recording capabilities, and image data of the glottis was gathered for creating an AI model. Data preprocessing and annotation of the vocal cords, epiglottis, and glottis were performed, and human annotation of the vocal cords, epiglottis, and glottis was carried out. Based on the AI's determinations, anatomical structures were color-coded for identification.

The recognition accuracy of the epiglottis and vocal cords recognized by the AI model was 0.9516, which was over 95%. The AI successfully marked the glottis, epiglottis, and vocal cords during the tracheal intubation process. These markings significantly aided in the visual identification of the respective structures with an accuracy of more than 95%. The AI demonstrated the ability to recognize the epiglottis, vocal cords, and glottis using an image recognition model of a manikin.

## Introduction

Conventional endotracheal intubation, even when performed by experienced practitioners, can make it difficult to obtain an adequate laryngeal view, and maneuvering with such limited visibility may result in dental trauma and oral injury [1-3]. Over the past two decades, video laryngoscopes equipped with digital cameras and screens for real-time laryngeal viewing during tracheal intubation have rapidly gained popularity. In certain situations, video laryngoscopes are emerging as standard tools due to their higher success rates and improved safety for tracheal intubation compared to conventional rigid laryngoscopes such as the Macintosh type. However, despite the use of video laryngoscopy, limited visibility and difficulties in discerning the structures in the laryngeal region may lead to failed tracheal intubation. The crucial point is that, whether using a direct laryngoscope or a video laryngoscope, accurate visual recognition of the larynx, especially the vocal cords, is paramount.

In recent years, the integration of artificial intelligence (AI) into image recognition has shown promising results in various medical fields [4,5]. The real-time and precise recognition of vocal cords and structures by AI during the procedure could serve as a valuable and promising support tool for tracheal intubation. However, there are few reports on methods that utilize AI to ensure accurate glottic view recognition. Biro et al. used an image analysis system to identify the larynx using an airway manikin. However, the details have not yet been disclosed [6]. This study aimed to explore the feasibility of an AI model that accurately recognizes the larynx on laryngoscopy images using an airway manikin for tracheal intubation training.

This article was previously posted to the Research Square preprint server on January 24, 2024.

## Technical report

This study was conducted with the approval of our hospital's (St. Marianna University School of Medicine) ethics committee. Tracheal intubation was performed using a manikin (MW13 Difficult Airway Management Simulator-Training Model; Kyoto Kagaku Company Limited, Kyoto, Japan) with a bronchoscope equipped with recording capabilities (BlueFire Fiberscope; BlueFire Limited, Shenzhen, China). The video data were preprocessed by dividing them into still images (640 × 480 dpi). The processing speed is approximately five frames per second. Human annotations were made on the preprocessed image data by specifying the bounding boxes and segmentations of the vocal cords, epiglottis, and glottis.

A combined model leveraging the strengths of YOLO (You Only Look Once) and UNet has been developed for enhanced decision-making in medical imaging [7]. YOLO is a state-of-the-art deep learning model known for its real-time object detection capabilities. It can efficiently identify and localize objects within an image, making it highly suitable for applications requiring instantaneous recognition, such as detecting cars or pedestrians in video streams. On the other hand, UNet is a highly effective model for image segmentation, particularly in medical image analysis. It excels in delineating specific structures within medical images, such as organs in MRI or CT scans, providing precise contouring essential for medical diagnosis and treatment planning. The integration of YOLO and UNet combines the rapid object detection capabilities of YOLO with the detailed segmentation prowess of UNet. This hybrid model can simultaneously detect and accurately outline specific regions of interest. For instance, in a medical setting, this model can be utilized to scan a patient’s body in real-time, promptly identifying the presence of a tumor while also delineating its precise shape and boundaries. In summary, the YOLO + UNet combined model capitalizes on the real-time detection efficiency of YOLO and the segmentation accuracy of UNet, making it a powerful tool for comprehensive and precise analysis in medical imaging.

This model enables the identification of an object’s location with high accuracy in a short time. In this study, specific regions of interest for the epiglottis, vocal cords, and trachea were set to recognize these anatomical structures using YOLO. On the other hand, UNet is an algorithm used to estimate the location of objects and is capable of real-time processing with a limited amount of training data. This model has been applied to the analysis of medical images. In this study, UNet was utilized to recognize the epiglottis, vocal cords, and trachea by freehand marking of these structures.

Based on the AI determinations, the epiglottis, vocal cords, and glottis are marked in blue, green, and red, respectively. We assessed the accuracy of visual determinations based solely on these markings. An intersection over union (IoU) was used for accuracy validation. The IoU is an accuracy metric used in tasks such as object detection and segmentation. The IoU evaluates the extent to which the predicted bounding box or segmentation mask overlaps with an actual target. The IoU of the predicted bounding box and the bounding box of the actual target were calculated; the IoU values ranged from 0 to 1, with values closer to 1 indicating that the prediction was more accurate.

**Figure 1:**
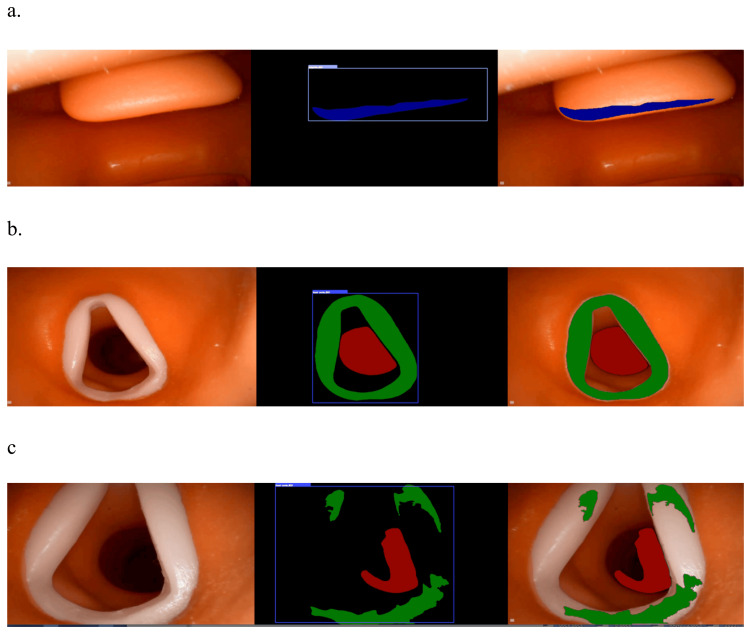
The AI delineated the epiglottis in blue, vocal cords in green, and glottis (tube insertion site) in red (a) Image of the vocal cords and glottis marked on a manikin: vocal cords are in green, glottis in red. (b) Image showing markings on the epiglottis of a manikin. The epiglottis is shown in blue. (c) Patterns that were poorly recognized due to light reflection and angle on markings of the manikin.

In this study, 920 images were collected, of which 54 images with clear depictions of anatomical structures were evaluated. The AI model underwent 100 training iterations. The accuracy (IoU) determined by the AI model was 0.9516, which exceeds 95%. The training process took 961.62 s, with five training cycles. The evaluation time per image was 0.1403 s, and the total evaluation time was 7.576 s. The output resolution was 680 × 480 dpi (input resolution, 640 × 480 dpi). In some instances, the model responded to unrelated regions. Throughout the tracheal intubation procedure, the AI delineated the epiglottis in blue, the vocal cords in green, and the glottis (tube insertion site) in red. This color coding facilitated visual identification of the respective structures with more than 95% accuracy (Figure 1).

## Discussion

This study demonstrated that AI could recognize the epiglottis, vocal cords, and glottis with greater than 95% accuracy on a model of the human body and that it was possible to visually determine anatomical structures by marking videos with AI.

The AI model was constructed using a combination of object detection (YOLO) and region extraction (UNet). Although the combined model is slower than the single model, it is expected to improve the accuracy by reducing the responses to irrelevant parts. In this study, the accuracy was close to 100% because a human model was used and there were no anomalies in the shape of the epiglottis or vocal cord. However, when the model darkened due to light reflection or light not reaching the model, determination became temporarily difficult. As there are many irregularities in the anatomical structures of the epiglottis and vocal cords, it is assumed that a large amount of teaching data will be required to build an AI model and that it will be necessary to consider alternative models for region detection. The speed of marking by the AI in the video could be depicted at a level that was comfortable for the person performing tracheal intubation.

In recent years, robot-assisted systems with specialized equipment and cameras, teleoperated robots, and automated robots that use AI and advanced algorithms to automate the entire endotracheal intubation process have been reported for airway management [8-10]. These remote robot-assisted tracheal intubation systems need to incorporate highly accurate airway recognition software to clarify the anatomical orientation. Cheng et al. developed a robot prototype called IntuBot in 2018 that aims to automate the intubation procedure [11]. In the area of automated intubation robotic endoscopy with airway imaging, Myers et al. conducted a proof-of-concept study using a manikin to explore the potential of a robotic endoscopy (REALITI) system via automated laryngeal imaging for tracheal intubation [12]. It consists of a video endoscope that allows manual control of the endoscope tip via a joystick and a flexible bronchoscope. It also incorporates image recognition software that allows the tip to move automatically toward the glottis when certain anatomical features are detected. The AI model used to determine the vocal folds in this software was built using a single model, which may not have been accurate. The use of a composite model, such as that used in this study, may enable a more accurate determination. In the future, many tracheal intubation devices are expected to be equipped with AI-based airway recognition systems. Recognition software development requires improved detection methods and a large amount of supervised data.

This study has several limitations. First, there was no irregularity in the structure of the object in this study because the marking was performed on a single manikin. Second, the accuracy of the image recognition model is verified using the same human model. Third, the state of the secretions and mucous membranes differs from that of the living body. Although there are few differences in the structures of characteristic anatomy, such as the vocal cords and epiglottis, among races and sexes, the condition of the oral cavity in the airway (bleeding and tumor) and the light reflection of mucous membranes and secretions may reduce the recognition accuracy of AI. To construct an image recognition model that can respond to individual differences in anatomical structures and diverse conditions of the living body, it is necessary to build an AI based on a large amount of data and human annotation.

## Conclusions

Using an image recognition model of the human anatomy, AI successfully identified the epiglottis, vocal cords, and glottis. If AI can accurately recognize and guide the viewpoint and route of tracheal intubation, it could contribute to safer intubation procedures. Anatomical guidance provided by AI has the potential to assist unskilled personnel and enable remote-controlled intubation. The integration of video laryngoscopes and automated intubation robots with AI-based airway guidance is anticipated in the future.
